# Reduction of depressive symptoms during inpatient treatment is not associated with changes in heart rate variability

**DOI:** 10.1371/journal.pone.0248686

**Published:** 2021-03-23

**Authors:** Sabrina Neyer, Michael Witthöft, Mark Cropley, Markus Pawelzik, Ricardo Gregorio Lugo, Stefan Sütterlin

**Affiliations:** 1 Eos-Klinik, Münster, Germany; 2 Department for Clinical Psychology, Psychotherapy and Experimental Psychopathology, University of Mainz, Mainz, Germany; 3 School of Psychology, University of Surrey, Guildford, United Kingdom; 4 Department for Information Security and Communication Technology, Norwegian University of Science and Technology, Gjøvik, Norway; 5 Faculty for Health and Welfare Sciences, Østfold University College, Halden, Norway; 6 Division of Clinical Neuroscience, Oslo University Hospital, Oslo, Norway; Erasmus Medical Center, NETHERLANDS

## Abstract

Vagally mediated heart rate variability (HRV) is a psychophysiological indicator of mental and physical health. Limited research suggests there is reduced vagal activity and resulting lower HRV in patients with Major Depressive Disorder (MDD); however little is actually known about the association between HRV and symptoms of depression and whether the association mirrors symptom improvement following psychotherapy. The aim of this study was to investigate the association between antidepressant therapy, symptom change and HRV in 50 inpatients (68% females; 17–68 years) with a diagnosis of MDD. Severity of depressive symptoms was assessed by self-report (Beck Depression Inventory II) and the Hamilton Rating Scale of Depression. Measures of vagally mediated HRV (root mean square of successive differences and high-frequency) were assessed at multiple measurement points before and after inpatient psychotherapeutic and psychiatric treatment. Results showed an expected negative correlation between HRV and depressive symptoms at intake. Depressive symptoms improved (d = 0.84) without corresponding change in HRV, demonstrating a de-coupling between this psychophysiological indicator and symptom severity. To our knowledge, this study is the first to examine an association between HRV and depressive symptoms before and after psychotherapy. The observed de-coupling of depression and HRV, and its methodological implications for future research are discussed.

## Introduction

MDD is one of the most common, and highly debilitating mental disorders, affecting an estimated 264 million people in 2020 [1]. MDD is typically associated with a significant reduction in quality of life [2, 3] and several physical illnesses especially cardiovascular diseases (CVD) [4–8]. To optimize the treatment for both conditions it is important to learn more about the biological aspects of MDD and its association with the cardiovascular system.

### HRV and autonomic nervous system (ANS)

One relevant indicator for psychopathological abnormalities is autonomic imbalance [9], which is reflected in a reduced heart rate variability (HRV) [10]. HRV is the variation of the period between two consecutive heartbeats over time. It indicates the adaptivity of the heartbeat to changing inner and outer conditions and thus reflects the adaptivity of the ANS [11]. Sympathetic and parasympathetic neurons innervate the heart via the vagus nerve and the stellate ganglion [10]. In their model of neurovisceral integration, Thayer and colleagues describe HRV in a dynamical systems framework [12, 13]. Within this model, cognitive, emotional, behavioral and physiological responses are regulated and reflected by excitatory and inhibitory innervation of the heart [14, 15]. A low adaptivity is an indicator of a relative sympathetic dominance and relative restricted parasympathetic tone. Low vagally mediated HRV has been associated with all-cause mortality [16–18], increased susceptibility to stress, emotional instability [19] and increased risk of cardiovascular diseases and mental disorders (e.g. depression) [17, 20].

### HRV and depression therapy

The results of two meta-analyses of MDD patients free of heart disease suggest that MDD patients have a reduced HRV compared to healthy controls [21, 22]. Furthermore, the severity of depressive symptoms appears to be inversely related to HRV and this association does not seem to be attributable to medication side effects [21].

Psychotherapy reduces depressive symptoms (g = 0.31) [23], but its effect on HRV remains unclear. Carney and colleagues [24] reported that severely depressed CVD patients benefit from cognitive behavioral psychotherapy (CBT) via depressive symptom reduction, heart rate reduction and increased HRV. In addition, a greater treatment response in depression was found to be associated with an increase in vagally mediated cardiac variability following acupuncture treatment [25]. It is worth noting that the sample was homogenous and does not represent a naturalistic clinical depressive sample. Nonetheless, Kim and colleagues [26] reported a significant improvement in HRV after a successful CBT/ meditation intervention in physically healthy depressed patients but not for CBT alone.

In contrast to the aforementioned findings, there are also indications that HRV may not significantly change following interventions despite the observed improvement in self-reported depressive symptoms. Carney and colleagues reported that moderately or mildly depressed CVD patients show reduced depressive symptoms without concomitant changes in HRV following CBT [24]. Wheeler and colleagues [27] showed that a mindfulness based cognitive therapy can reduce depressive symptoms but did not affect HRV. Similarly, Brunoni and colleagues [28] found no improvement in HRV after a non-pharmacological (transcranial direct current stimulation) or pharmacological (Selective Serotonin Reuptake Inhibitor, SSRI) treatment of unipolar depression.

Besides these inconclusive results, the effects and side effects of antidepressant medication on HRV similarly shows contradictory findings [22, 29–35]. A meta-analysis by Kemp and colleagues [22] concluded that typically used antidepressants had no significant impact on HRV, apart from tricyclic antidepressants that decreased HRV. However, individual studies suggest that there could be a tendency that antidepressants lower HRV [30, 31, 36, 37].

### Research gap

The question of whether HRV could represent a biomarker not only for depression before treatment, but also for therapeutic change remains open. One possible explanation for the inconsistent findings may be that even after successful intervention, psychophysiological correlates of depression remain stable [38–40]. Greenberg and colleagues suggest that this observed lack of HRV improvement in depressive patients might be one reason for the high relapse rates [41]. Those studies, reporting no changes in HRV following symptom reduction, explained this absence mostly with a delay of physiological changes following psychological changes [32]. Overall, the evidence that psychotherapy might be able to increase HRV in patients with MDD remains ambiguous.

### Methodological problems of HRV measurements

Regardless of the inconsistent findings, the current available studies contain some methodological shortcomings. Firstly, they focus on too specific samples (often persons with mild levels of depression and without any medication) which mostly do not reflect the regular patients in clinical psychotherapy settings [25, 26]. Therefore, the generalizability of these findings to patients with severe depressive disorders is limited. Second, the few existing studies comparing pre- and post-intervention HRV do not show any intervention effects between depressive symptom reduction and HRV values [28, 42]. Third, there are only a few studies comparing pre and post intervention HRV values but all of them have used single short-term recordings for each timepoint [25, 26, 28]. This is problematic because HRV values have been shown to be highly state-dependent due to situational context factors [43, 44] and they tend to display large day-to-day random variations, which makes it difficult to discover intervention effects within individuals [45]. Fourth, it is difficult to compare the results of different studies because of the use of different HRV parameters, measurement hard- and software across and within each study [46, 47].

### Contribution

The purpose of the present study is to investigate the association between HRV and depressive symptoms before and after an intensive psychiatric psychotherapeutic treatment. From a methodological perspective, we considered it important to achieve a robust HRV measurement by using multiple measurements to compensate for situational variance [43, 44]. In addition, we sampled MDD patients in a naturalistic inpatient setting.

## Methods

### Participants

The sample consisted of 50 inpatients (*N* = 34 females, *N* = 16 males) admitted for psychotherapy in a German psychosomatic hospital, with a *Mea*n age of 39.51 years (*SD* = 14.97; *Range* = 17.5–67.8 years). Twenty-six percent of the participants identified themselves as smokers, smoking an average of 13.75 cigarettes per day (*SD* = 12.48; min<1; max = 40).

The admission criteria for clinical treatment were serious depressive symptoms or serious social impairment so that everyday requirements could no longer be met; patients experiencing treatment resistant depression, or where an outpatient therapy did not lead to an improvement of depressive symptoms [48]. The participants’ diagnoses were assessed through a structured clinical interview (SCID I, II) [49–51]. Inclusion criteria for the present study was a diagnosed MDD. Exclusion criteria were evidence of comorbid excessive substance or alcohol use, psychosis, autoimmune-thyroiditis, anorexia nervosa, BMI<18.0, respiratory, hormone or heart diseases. Seventy-six percent of the inpatient sample fulfilled the criteria for at least one additional comorbid mental disorder. The number of comorbid diagnoses ranged from 0 to 6 (*M* = 1.86, *SD* = 1.48). Table 1 shows the distribution of comorbid diagnoses.

**Table 1:** Comorbid disorders.

	N	%
Personality Disorders	29	58
Eating Disorders	13	26
Posttraumatic Stress Disorder	8	16
Somatoform Disorders	6	12
Anxiety Disorders	6	12
Others	5	10
No comorbidities	12	24
	50	100

*Note*. Number of patients with a comorbid diagnosed disorder (e.g. attention deficit hyperactive disorder or inorganic sleeping disorders).

Sixty-eight percent of patients were prescribed antidepressant medication during treatment. Average onset of depression was 10.72 years before their current inpatient treatment (*SD* = 9.59; *Rang*e = 1–35 years). Fifty-eight percent of the participants had been previously hospitalized, while for 30% this was their first time as inpatients (for the remaining 12% this information was missing). The *Mean* number of previous inpatient therapies was 1.42 (*SD* = 1.47; *Range* = 0–5 previous inpatient therapies). This study was approved by the Ethics Committee of the “Medical Association Westfalen-Lippe” and written informed consent was obtained from all participants prior to data collection.

### Design

This study utilized a longitudinal naturalistic pre-post-design. All patients completed routine computer based self-report questionnaires during their first and last week of inpatient therapy, while a clinical psychologist conducted the Hamilton-Interview during the first and last week. The patients stayed between 6 and 12 weeks (*M* = 8.80; *SD* = 2.5) as inpatients and attended an individual psychotherapy session five times per week and at least one group therapy per weekday (e.g. Mindfulness based therapy, Mentalization based Therapy, social skills training, Psychoeducation for Depressive Disorders). Psychopharmacotherapy prescriptions were reviewed at least once a week and adapted if necessary. The CBT interventions differed between patients to accommodate for the heterogeneity of depressive disorders and symptoms. HRV assessment took place during the first and last week of therapy on three days (normally Monday, Wednesday and Friday morning between 9 and 11 am). Three assessments at the beginning and at the end of therapy were used to reduce the high impact of situational confounders and to increase the transsituational variance from about 49% following one-time assessments up to 75% for two or three assessments [43].

### Instruments

#### Short term HRV assessment

At the beginning of each individual HRV assessment, the experimenter checked whether the following exclusion criteria were met: refraining from smoking or drinking caffeinated beverages at least three hours before the measurement and not participating in morning exercise on measurement days. The time period of three hours was based on the daily clinical routine and also applied in comparable studies [see 52–54]. If necessary, the assessment was postponed to the following day.

The experimenter explained the procedure and gave a short information about basic functions of the ANS. Participants were requested to switch off their mobile phones before measurement started. The experimenter assisted with administering the electrocardiogram (ECG) electrodes (disposable ECG-electrodes with fluidity impairment foamed material from Dahlhausen, Köln, Germany) correctly (Einthoven’s triangle: Lead III). After administering the measurement hardware and before starting the recording, the experimenter checked if the equipment worked properly and asked if the patient felt well enough to proceed. After this short stabilization period [55, 56] the actual HRV measurement began. During each measurement the patient was asked to sit still in a comfortable chair and breathe normally for the next five minutes to assess the ECG baseline. The electrocardiogram [57] was recorded at a 500 Hz sampling rate. The experimenter was seated outside in front of the room during the recording.

#### Hamilton rating scale of depression

The Hamilton Rating Scale of Depression (HRSD) is an assessment tool to record the severity of depressive symptoms [58, 59]. It consists of 24 items, scored from 0 to 4. It is sensitive to change and therefore suitable for use in clinical trials. The internal consistency (Cronbach’s alpha) of the HRSD at intake and at discharge was adequate to very good: HRSD_Intake = .77 (*N* = 49); HRSD_Discharge = .91 (*N* = 37). Both are comparable to a previous validation of the German HRSD version [60].

#### Beck Depression Inventory-II

The Beck Depression Inventory-II (BDI-II) is a 21-item self-report questionnaire used to assess the severity of depressive symptoms [61, 62]. Each item consists of four response statements that are rated from 0 to 3 representing ascending severity of depressive symptoms. A total value of 0–9 indicates minimal depression, 10–18 indicates mild depression, 19–29 indicates moderate depression and 30–63 indicates severe depression. The internal consistency (Cronbach’s alpha) of the BDI-II at intake and discharge were very good: BDI-II_Intake = .93, BDI-II_Discharge = .96 (N = 50) and these values are comparable to a published study conducted with the German BDI-II version [63].

### Data reduction

ARTiiFACT software [64] was used to extract QRS complexes, determine interbeat intervals, and to detect and correct the raw ECG. In line with common research standards, HRV measures indicating vagally mediated HRV were extracted. For the time value, we used the Root Mean Square of Successive Differences (RMSSD) [10]. RMSSD reflects the variance of successive beat to beat intervals and is a reliable indicator for vagal activity during 5-minute short-term recordings with spontaneous breathing [65]. In addition, the RMSSD appears to be more robust against state influences compared to other HRV values. As a frequency measure, power of the high frequency band (HF power, 0.15–0.4Hz) was derived [10]. HF power is also indicative of parasympathetic activity [65, 66].

### Statistical analyses

Prior to analysis, all variables were checked for accuracy of data entry and missing values. Little’s MCAR Test for all psychometric and biometric data showed a statistically non-significant result (χ2(140) = 118.71, *p* = .91) indicating that values missing completely at random could be inferred [67].

All variables were checked for univariate outliers by identifying cases with *z*-score above 3.29 or below -3.29, and these were dealt with by deletion. Consequently, two individual’s HRV measurements (*HF_T1_Intake* and *RMSSD_T1_Intake*) at intake, and five individual’s measurement at discharge (*HF_T1_Discharge*, *RMSSD_T1_Discharge*, *HF_T2_Discharge*, *RMSSD_T2_Discharge* and *HF_T3_Discharge*) were identified as outliers and deleted without replacement. Other missing values (in total: 40 values) result from premature discharge, non- compliance or technical problems.

The HRV variable HF was normalized via log(n)-transformation [68]. All other variables were normally distributed without transformation. Normalization might have an impact on the data analysis, so we checked all analyses without normalization of data. No significant change of results occurred within this additional step of analysis. *Mean* HRV indices were calculated for all three respective measurement points at intake (RMSSD_Intake, lnHF_Intake) and all three respective measurement points at discharge (RMSSD_Discharge, lnHF_Discharge).

Cronbach’s alpha was calculated to test the reliability of all four indices (RMSSD_Intake, lnHF_Intake, RMSSD_Discharge and lnHF_Discharge) based on the respective three measurement points. Cronbach’s alpha values were.73-.89 for each index and therefore can be considered good to very good. The associations between HRV variables and questionnaire measurements (intake, discharge) were assessed using Pearson’s correlations and *T*-Tests. Statistical analysis was performed using SPSS 25 [69]

## Results

### Descriptive statistics

We examined the association between MDD symptoms and HRV before and after inpatient therapy. The descriptive statistics and psychometric results at intake and discharge are presented in Table 2 including *Mean* intake HRV and *Mean* discharge HRV indices.

**Table 2:** Summary of descriptive psychometrics and HRV values.

		*N*	*M*	*SD*	Min	Max
Descriptive data and symptom severeness	Age (years)	50	39.51	14.97	17.49	67.75
	BMI	50	24.26	3.97	18.0	34.6
	Duration of CBT (weeks)	50	8.8	2.5	6	12
	Previous stationary therapies	50	1.42	1.47	0	5
	HRSD Intake	45	23.51	7.15	10.00	37.00
	HRSD Discharge	40	14.33	10.49	1.00	49.00
	BDI-II Intake	49	28.41	12.94	5.00	52.00
	BDI-II Discharge	50	16.70	14.78	0.00	53.00
HRV values	RMSSD T1 Intake (ms)	49	22.17	11.37	3.75	43.97
	RMSSD T2 Intake (ms)	49	21.92	14.43	2.76	63.41
	RMSSD T3 Intake (ms)	48	21.08	11.77	5.76	48.34
	RMSSD Mean Intake (ms)	50	21.62	11.34	5.38	49.42
	RMSSD T1 Discharge (ms)	49	22.03	13.38	4.63	62.76
	RMSSD T2 Discharge (ms)	49	20.97	11.64	4.92	54.03
	RMSSD T3 Discharge (ms)	42	20.21	11.20	3.99	53.21
	RMSSD Mean Discharge (ms)	50	21.62	10.56	4.99	48.84
	Ln HF_T1_Intake (ms^2^)	49	3.96	1.30	0.76	6.12
	Ln HF_T2_Intake (ms^2^)	49	3.89	1.38	-0.11	6.08
	Ln HF_T3_Intake (ms^2^)	48	3.89	1.36	1.11	6.02
	Ln HF_Mean Intake (ms^2^)	50	3.89	1.22	1.34	5.74
	Ln HF_T1_Discharge (ms^2^)	49	3.96	1.24	0.73	6.28
	Ln HF_T2_Discharge (ms^2^)	49	3.77	1.20	0.89	5.88
	Ln HF_T3_Discharge (ms^2^)	41	3.80	1.18	0.99	5.92
	Ln HF_Mean_Discharge (ms^2^)	50	3.90	1.06	0.93	5.88

*Note*. Abbreviations: M = Mean, SD = Standard deviation, Min = Minimum, Max = Maximum, HRSD = Sum score of Hamilton Rating Scale of Depression, BDI-II = Sum score of Beck Depression Inventory, RMSSD = Root Mean Square of Successive Differences, Ln HF = Power of High Frequency Band.

The HRV values at intake were comparable to previous studies with MDD patients (e.g. RMSSD *M* = 27.16 [70] or RMSSD *M* = 23.50 [29]) and notably lower than normed HRV values of healthy persons (RMSSD *M* = 42, *SD* = 15 [71]). The intake self-reported data was comparable to other studies investigating psychotherapy outcomes of CBT after an MDD inpatient treatment [24, 72].

### Correlation between HRV values and psychometric results

There was a significant positive correlation (Pearson’s r) between the two psychometric indices (BDI-II & HRSD) (for all min. *r*>.37, *p* < .05) and a significant positive correlation between the two HRV indices (RMSSD & HF) (for all *r*>.49, *p* < .001) (see Table 3).

**Table 3:** Correlation between HRV values and depression symptom severeness.

			HRV values			Symptom severeness				
			RMSSD Discharge (ms)	Ln HF Intake (ms^2^)	Ln HF Discharge (ms^2^)	BDI-II Intake	BDI-II Discharge	HRSD Intake	HRSD Discharge	Stationary Therapies
HRV values	RMSSD Intake (ms)	*R*	.522***	.872***	.586***	-.293*	-.186	-.352*	-.270	-.303*
		*P*	**< .001**	**< .001**	**< .001**	**.041**	.200	**.019**	.111	**.049**
		*N*	50	50	50	49	49	44	36	43
	RMSSD Discharge (ms)	*R*		.486***	.811***	-.125	-.055	-.133	-.180	-.208
		*P*		**< .001**	**< .001**	.391	.708	.391	.294	.181
		*N*		50	50	49	49	44	36	43
	Ln HF Intake (ms^2^)	*R*			.697***	-.209	-.156	-.360*	-.182	-.363*
		*P*			.001	.150	.285	**.017**	.288	**.017**
		*N*			50	49	49	44	36	43
	Ln HF Discharge (ms^2^)	*R*				-.102	-.076	-.185	-.121	-.304*
		*P*				.487	.603	.229	.482	**.004**
		*N*				49	49	44	36	43
Symptom values	BDI-II Intake	*R*					.605***	.714***	.529**	.434**
		*P*					**< .001**	**< .001**	**.001**	**.004**
		*N*					48	43	36	42
	BDI-II Discharge	*R*						.483**	.738***	.366*
		*P*						**.001**	**< .001**	**.017**
		*N*						43	35	42
	HRSD Intake	*R*							.369*	.342*
		*P*							**.038**	**.033**
		*N*							32	39
	HRSD Discharge	*R*								.312
		*P*								.082
		*N*								32

*Note*: Abbreviations: HRSD = Sum score of Hamilton Rating Scale of Depression, BDI-II = Sum score of Beck Depression Inventory, RMSSD = Root Mean Square of Successive Differences, Ln HF = Power of High Frequency Band.

Significance (two-tailed),

* *p* < .05.

** *p* < .01.

*** *p* < .001.

**Fig 1:**
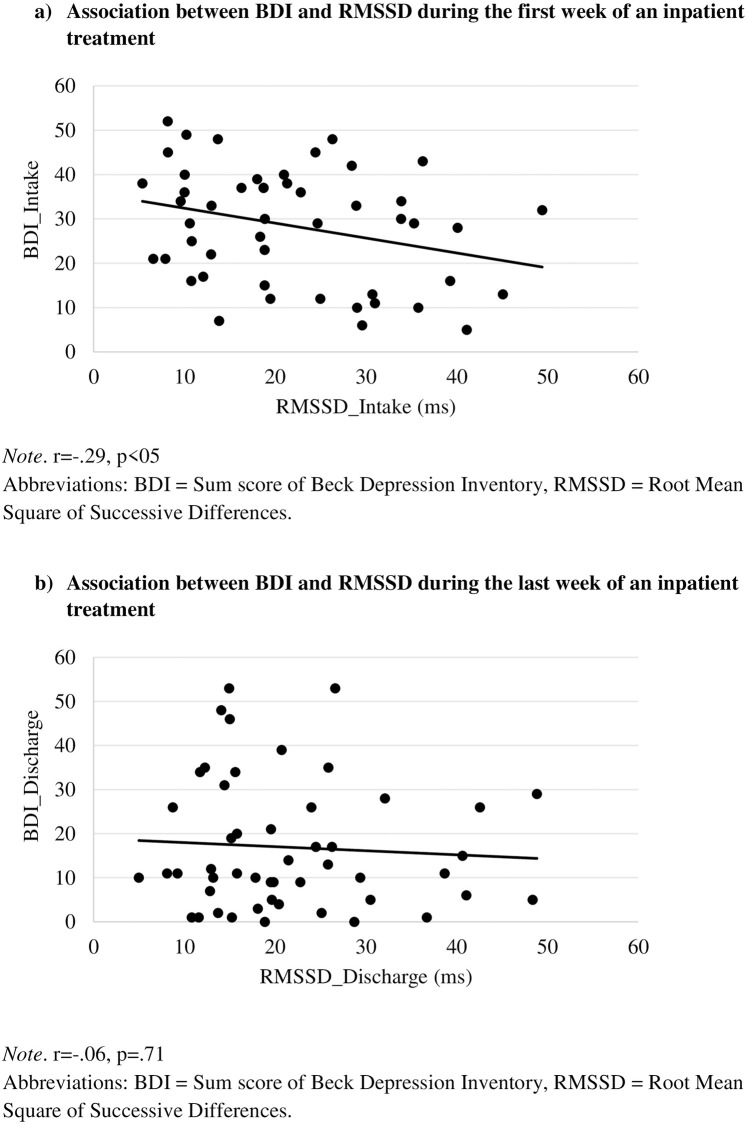
a) Association between BDI-II and RMSSD during the first week of an inpatient treatment. *r* = -.29, *p*<05. b) Association between BDI-II and RMSSD during the last week of an inpatient treatment. *r* = -.06, *p* = .71. Abbreviations: BDI-II = Sum score of Beck Depression Inventory, RMSSD = Root Mean Square of Successive Differences.

There was a significant negative correlation between RMSSD_Intake and psychometric intake measurements (*r* = -.29, *p* < .05). Ln_HF_Intake only showed a significant correlation with HRSD_Intake. At discharge there was no significant correlation between HRV and psychometric indices. None of the aforementioned significant correlations reached significance at discharge (e.g. see Fig 1).

There was no significant correlation between HRV-change indices and symptom-reduction indices (discharge minus intake values) (for all *p*>.16). The change of intake HRV indices and discharge HRV indices was not significantly associated with symptom-reduction variables either (for all *p*>.20).

Symptom severity was significantly positively correlated with the duration of psychotherapy (Intake: *r* = .56, *p* < .001 and Discharge: *r* = .38, *p* < .01). *T*-Test comparisons of subgroups showed that smokers and non-smokers did not differ on depression severity (e.g. Smokers: BDI-II_Intake *M* = 33.83, *SD* = 16.45; Nonsmokers: BDI-II_Intake *M* = 26.65, *SD* = 11.30; *T* = 1.70, *p* = .10) or HRV values (Smokers: RMSSD_Intake *M* = 20.53, *SD* = 13.62; Nonsmokers: RMSSD_Intake *M* = 22.01, *SD* = 10.61; *T* = .40, *p* = .69). There was no significant correlation between smoking behavior and HRV (RMSSD_Intake *r* = -.06; *p* = .69; RMSSD_Discharge *r* = .03, *p* = .86) or Depression values (BDI-II_Intake *r* = .16; *p* = .27, BDI-II_discharge *r* = .24, *p* = .10). T-Test comparisons of subgroups showed that normal weight (female BMI 18–24, male 19–25) persons did not differ from overweight (female BMI>24, male BMI>25) persons in depression severity or HRV values (for all comparisons *T*<1.24, *p*>.22).

The repetition of all calculations with only the first measurement at intake and the first measurement at discharge showed no significant association between physiological and psychological values at intake or at discharge. Consistent with the results based on *Mean* HRV values, there were no significant differences between admission and discharge measurements. Other indicators (Heart Rate, SDNN, pNN50 or LF/HF Ratio) were calculated but did not lead to different results and did not change between admission and discharge (see S1 and S2 Tables).

### Symptom reduction

All psychometric questionnaires for the assessment of MDD symptoms showed significant reductions (see Table 4). On average, the BDI-II_Discharge values were reduced by 11 BDI-II-points compared to BDI-II_Intake. Following therapy, 20% of patients continued to display severe BDI-II symptom severity, 16% as moderate, with 60% of patients demonstrating no, minimal or mild depressive symptoms. The HRSD_Discharge values were reduced on average by 7 points.

**Table 4:** Paired T-Tests for depression and HRV indices at intake and discharge of psychotherapy.

	N	Intake	Discharge	Df	t	P	d
HRSD	36	23.39 (7.24)	14.14 (10.93)	35	5.11	< .001	.874
BDI-II	49	28.41 (12.94)	16.84 (14.90)	48	6.58	< .001	.837
RMSSD (ms)	50	21.62 (11.34)	21.62 (10.56)	49	.00	.997	.0001
Ln HF (ms^2^)	50	3.96 (1.30)	3.96 (1.22)	49	-.06	.95	-.007

*Note*. Abbreviations: HRSD = Sum score of Hamilton Rating Scale of Depression, BDI-II = Sum score of Beck Depression Inventory, RMSSD = Root Mean Square of Successive Differences, Ln HF Band = Power of High Frequency Band.

The HRV indices showed no significant changes after inpatient psychotherapy and the *SD*s did not vary between intake and discharge (for all indices *p*>.95). Nevertheless, intraindividual *SD*s showed fluctuations within the three measurements at intake (RMSSD: *Mean_SD* = 5.51; *SD_SD* = 4.01; ln_HF: *Mean_SD* = .58; *SD_SD* = .39) and within the three measurements at discharge (RMSSD: *Mean_SD* = 6.87, *SD_SD* = 4.45; ln_HF: *Mean_SD* = .64; *SD_SD* = .46). Paired *T*-Tests showed no significant difference between intake variability of HRV values and discharge variability of HRV values (for all intraindividual HRV values *p*>.45).

### Gender analysis

A *T*-Test of HRSD showed that women (HRSD_Intake *M* = 25.23; *SD* = 7.12) had higher HRSD depression scores than men (HRSD_Intake *M* = 20.07; *SD* = 6.05) at the beginning of psychotherapy (*t*(43) = 2.41, *p* = .02). All other psychometrics showed similar differences between men and women at intake and discharge. There was no significant difference between men and women in HRV values (for all HRV values *p*>.12).

## Discussion

The aim of the present study was to investigate—by applying robust HRV estimations—the association between HRV and depressive symptoms before and after an intensive inpatient treatment in a sample of MDD inpatients. This study combines self-reported, third-party and psychophysiological data and is one of the first based on a naturalistic sample. It is also one of the first studies to utilize average multiple HRV assessments to obtain trait-like characteristics in a clinical sample.

### Association between MDD and HRV at intake

In line with previous controlled studies patients with MDD showed lower than average HRV indices at intake [73]. Levels of depression were comparable to published inpatient samples at intake for CBT [29, 72, 74]. As expected, a positive association between symptom severity and duration of psychotherapy was observed. At the time of intake before treatment onset, MDD symptom severity and HRV indices were negatively associated, replicating previous clinical studies [22]. These findings support the notion that depressed patients show reduced vagal activation, underlining the role of vagally mediated HRV as a biomarker for mental health. The current state of research suggests different explanations for these findings at intake: It can be assumed that somatic symptoms of MDD (e.g. sleeping problems, changes in appetite, fatigue, pain) are more pronounced in severe depression and are associated with a decrease in HRV [75]. An alternative explanation could be the processing of socially threatening stimuli, which are perceived stronger by depressive persons and therefore lead to a stronger autonomous reaction [53]. Patients experience worries and hypervigilance have particularly severe difficulties in deactivating threatening stimuli [76]. This lack of inhibition leads to chronic overactivity of the sympathetic nervous system and reduced parasympathetic withdrawal, which both decrease the ability of ANS to adapt to inner and outer circumstances because of a defect in noradrenaline reuptake [77–79], resulting in lower HRV. An implication of sustained SNS overactivity is an autonomous imbalance with a relatively low parasympathetic activation associated with depressive symptoms and—in nonclinical samples—emotional dysregulation [80].

### No association between MDD and HRV at discharge

Following intervention, depressive symptoms showed significant reductions on all the self- and third-party assessments. However, the HRV indices as estimates of vagal activation remained unchanged. The significant association between HRV indices and MDD symptoms (self-reported and externally assessed) at the beginning of psychotherapy disappeared post-intervention. Thus, the symptom alleviation during treatment does not seem accompanied by a simultaneous improvement of HRV. The assumption that HRV might be a specific biomarker for current depressive symptoms cannot be supported in regards to post-treatment situations. In line with previous findings [22, 26, 32], our results rather suggest that inpatient CBT and psychiatric treatment significantly reduce depressive symptoms without changing short term HRV values at the same time.

One explanation for the dissociation between the longitudinal development of depression and HRV, might be that psychotherapy helps depressive patients gain more insight into dealing with depression, and helps them become more self-compassionate. Patients learn how to behave and think in different situations. HRV has been reported to reflect inhibitory and emotion regulatory capacity [15, 17]. It could be speculated that this capacity might be triggered by psychological interventions [81]. The assumption of a better use of regulatory cognitive strategies obtained during the psychotherapeutic treatment, i.e., a more efficient use of existing neural capacities, does not require the assumption of an increased HRV baseline. In line with this suggestion, Brunonni and colleagues [28] suggest that a reduced parasympathetic activity might be a trait factor for depression which explains the high relapse rates [82] rather than a state marker for depressive symptoms. Consequently, low resting HRV might not be a state-like indicator of a current depression level, but an endophenotype of the underlying vulnerability and thus persisting beyond successful treatment and symptom alleviation. In depressed persons, vulnerability and symptoms coincide. This view on HRV as a vulnerability marker is supported by evidence showing low HRV to be a risk factor for the later development of depression and a marker for various risk factors contributing to depression, such as dysfunctional emotion regulation or perseverative thinking [13, 83, 84]. In this sense, HRV should be seen as a transdiagnostic marker for stress and psychopathological vulnerability that can coincide with clinical manifestation in untreated individuals, but not as a specific biomarker for depression. This result is in line with Beauchaine and Thayer [9] who suggest that especially HF-HRV can be considered an “transdiagnostic biomarker of psychopathology” (p. 345). Apart from that the high portion of within-subject variance of HRV values also makes it difficult to detect changes in HRV values over time [44].

This explanatory approach in the tradition of vulnerability-stress-models remains to date speculative due to a lack of empirical evidence on patients’ pre-morbid HRV status. It is, however, compatible with the notion that low HRV is a risk factor for a wide range of mental disorders. Furthermore, it is compatible with the observation of successful symptom alleviation in the absence of HRV changes. The extent to which this explanation accounts for symptom alleviation can only be tested in conditions that are structurally (in terms of frequency, intensity and set-up) identical with the actual treatment condition.

Importantly, it should be mentioned that reduced HRV is not a specific feature of MDD but rather a transdiagnostic factor which relates to several stress-related states, conditions and behavioral factors as well as to medical conditions and antidepressant medication [85]. Psychiatric and psychotherapeutic interventions may therefore not be sufficient to change neurobiological processes, which may only take place after a global change in life and behavior. Despite the improved mood, there may still be unfavorable life-style factors (e.g. smoking, sleep disorders, lack of activities, overweight, etc.) that could explain the consistently reduced HRV values [86, 87]. This hypothesis is supported by the meta-analysis by Gan and colleagues [88] supporting the effects of unfavorable lifestyle factors on the risk of developing CVD.

With regard to specific lifestyle factors research shows that nicotine disturbs normal ANS functioning by increasing SNS activity and reducing PNS modulation [87]. Similar mechanisms have been shown for the impact of overweight [89]. Especially for depressed patients Harte and colleagues [90] showed that depressed smokers had significantly increased sympathetic tone which manifests in reduced HRV values compared to depressed non-smokers. In our sample, there were people who continued to have unfavorable lifestyle factors e.g. smoking. Since this change is not the main goal of inpatient therapy and a global change in lifestyle and behavior would only become apparent in the outpatient setting in the long term. HRV improvement might take more time because of the necessary neurological changes in the central autonomic network (CAN) [15].

Another possible explanation for the constantly reduced HRV values could be the intake of psychotropic drugs, which were prescribed, adjusted or discontinued individually throughout the therapy. There might be an association between HRV values and antidepressant medication which is responsible for the autonomic disbalance [42]. In addition, Licht and colleagues [32] found that HRV values were lower in people taking antidepressant medication compared to people without medication regardless of the success of the therapy. Furthermore, Brunoni and colleagues [28] found that HRV scores did not change following treatment with either a non-pharmacological (tDCS) or pharmacological (sertraline) intervention, nor did HRV increase with clinical response to treatment.

### Methodological considerations

The variability of HRV values is considerable between the various intraindividual measurement points. This instability of HRV short term recordings should not be confused with low reliability, as short-term recordings are considered to be quite accurate estimations of vagal activation [10, 91]. The temporal volatility rather reflects the imminent state characteristics of the ANS, and the vagal activity of depressive persons in particular. Consistently, Bertsch and colleagues have shown that the state dependent variance in single measurements is 49% and can be reduced to 25% by using multiple measurements [43]. Therefore it seems prudent to measure HRV from multiple measurements to reduce the situational influences, especially for naturalistic settings [43, 91, 92]. Especially for a clinical naturalistic sample, HRV measurement is influenced by multiple confounders, and at present, no clear guidelines or comparative values for depressive patients exist. This lack of comparable studies may be due to a considerable publication bias because we would expect more studies reporting no significant associations between MDD and HRV especially after therapy, or a higher variability within the different study findings. Another explanation could be the high effort to use a robust research design in a clinical naturalistic setting. In addition, the comparability of published HRV values between studies is difficult because there are a large number of different HRV indices with different implications and interpretations of each indices [93, 94].

### Limitations

When interpreting the findings of the present study, the following three limitations need to be considered. Firstly, the possible effects of antidepressant medication on HRV have been widely discussed in the current literature, without any overall agreement [22, 32, 37]. Due to the severity and duration of their disorders, patients within our dataset were prescribed antidepressant medication. In addition, adjustments and terminations of medication during inpatient therapy are common within this natural setting, and thus lead to changes in individual medication combinations. These factors could not be calculated as control variables since the respective subgroups were too small. However, potential interaction between antidepressant medication and HRV values cannot be ruled out.

Second, in addition to the diagnosis of depression, other mental illnesses, e.g. anxiety disorders [95] or personality disorders [96, 97] seem to be associated with reduced HRV values. Our sample consists of seriously depressed patients with a large variety of comorbidities, which may have affected the results. These possible effects cannot be statistically isolated due to the many combinations and resulting homogeneous sub-groups. Additionally, depression itself is a very heterogeneous disorder and strongly different subgroups exist [29] in our naturalistic sample recruited directly in a psychosomatic hospital. Lastly our study does not include a healthy control group, so that comparable studies (e.g. with normed values [71] or similar measurement and calculation methods) were used to classify the HRV values. Besides, the absolute HRV values are not of primary importance but rather the relationship between HRV and depression.

Third, although we controlled for the most significant situational confounders, we were not able to control for all possible confounders. For example, there is evidence that the menstrual cycle or Body Mass Index can affect HRV levels [98] or that ruminative thoughts might lower HRV [83]. We tried to minimize these effects with three measurements on different days but there might be still confounders impacting our findings.

### Future research

Future studies investigating the association between HRV and MDD before and after psychotherapy should consider subgroups within the (naturalistic) sample. The most relevant subgroups in this sense are based on comorbidities and medications. Regarding comorbidities, Kircanski and colleagues [99] showed that only in anxious depressed patients, HRV can predict the treatment outcome. Consequently, a three-group design (patients with depression, patients with comorbid depression and anxiety and patients with anxiety disorders only) with a large sample to separate these comorbidities, seems necessary. Regarding medications, despite a large evidence base, there are no clear conclusions of confounding effects on HRV measurement. It cannot be precluded that changes of the autonomic nervous system after a successful MDD therapy are masked by the effects of antidepressants. Consequently, a subgroup design considering the medications and including a control group without medications seems necessary.

From a methodological perspective, multiple HRV measurements should be used in future investigations to obtain more valid and reliable data compared to one-time measurements. Alternatively, long-term HRV measurements could be used to further analyze potential long-term effects. In addition, future studies could augment the study design with a follow-up measurement after discharge (e.g. six months later) to examine if there is a lag effect of HRV, at the end of therapy.

## Conclusion

The present study is among the first to examine HRV before and after a psychotherapy inpatient treatment in a naturalistic sample. By measuring HRV multiple times at intake and multiple times at discharge and considering situational factors, we collected reliable and valid psychophysiological data. In summary, we observed an association between MDD and HRV values at intake, but not at discharge, even though depressive symptoms improved significantly. Therefore, HRV does not appear to be suitable as a change-sensitive biomarker for depression. This means that even after successful psychotherapy, the autonomic imbalance remains the same and can still be treated as a risk factor for diseases like CVD or an additional depressive episode. For this reason, in addition to psychotherapy, behavioral change techniques should be promoted, that are known to have beneficial effects on the autonomic nervous system, e.g. physical exercise, smoking cessation and healthy eating.

## Supporting information

S1 Table Summary of additional descriptive HRV values. (DOCX)

S2 Table Paired T-Tests for additional HRV indices at intake and discharge of psychotherapy. (DOCX)

S1 Dataset (SAV)
