# Supplementary material for: Reduction of depressive symptoms during inpatient treatment is not associated with changes in heart rate variability
Source: PLoS One. 2021 Mar 23;16(3):e0248686. doi: 10.1371/journal.pone.0248686 (PMC7987172; doi:10.1371/journal.pone.0248686)
Supplement: S1 Table — (DOCX) [file pone.0248686.s001.docx]

| **S1 Table. Summary of additional descriptive HRV Values** | | | | | | | |
| --- | --- | --- | --- | --- | --- | --- | --- |
|  |  | *N* | *M* | *SD* | Min | Max |  |
| HRV Values | Heart Rate Intake | 50 | 80.55 | 12.76 | 56.52 | 118.05 |  |
|  | Heart Rate Discharge | 50 | 80.08 | 10.05 | 62.39 | 114.20 |  |
|  | SDNN Intake in ms | 50 | 36.00 | 14.23 | 12.64 | 76.72 |  |
|  | SDNN Discharge in ms | 50 | 36.96 | 14.42 | 15.28 | 86.47 |  |
|  | pNN50 Intake in % | 50 | 2.92 | 4.03 | 0.00 | 17.32 |  |
|  | pNN50 Discharge in % | 50 | 2.76 | 4.06 | 0.00 | 15.21 |  |
|  | LF/HF Ratio Intake in % | 50 | 4.28 | 5.21 | 0.58 | 34.48 |  |
|  | LF/HF Ratio Discharge in % | 50 | 3.58 | 3.20 | 0.33 | 16.74 |  |

*Note*. Abbreviations: M = Mean, SD = Standard deviation, Min = Minimum, Max = Maximum, ms = milli seconds, pNN50% = Percentage of successive RR intervals that differ by more than 50ms; SDNN = Standard deviation of interval between two normal R-peaks, LF/HF Ratio = Ration of relative power of the low-frequency band (0.04–0.15Hz) to relative power of the high-frequency band (0.15–0.4Hz).
