# Supplementary material for: Reduction of depressive symptoms during inpatient treatment is not associated with changes in heart rate variability
Source: PLoS One. 2021 Mar 23;16(3):e0248686. doi: 10.1371/journal.pone.0248686 (PMC7987172; doi:10.1371/journal.pone.0248686)
Supplement: S2 Table — (DOCX) [file pone.0248686.s002.docx]

**S2 Table. Paired T-Tests for additional HRV indices at Intake and Discharge of Psychotherapy**

|  | N | Intake | Discharge | Df | t | p | d |
| --- | --- | --- | --- | --- | --- | --- | --- |
| Heart Rate | 50 | 80.55 (12.76) | 80.08 (10.05) | 49 | .34 | .74 | -.04 |
| SDNN | 50 | 36.00 (14.23) | 36.96 (14.42) | 49 | -.60 | .55 | .09 |
| pNN50 | 50 | 2.92 (4.03) | 2.76 (4.06) | 49 | .27 | .79 | -.04 |
| LF/HF Ratio | 50 | 4.28 (5.21) | 3.58 (3.20) | 49 | 1.12 | .27 | -.14 |

*Note*. Abbreviations: pNN50% = Percentage of successive RR intervals that differ by more than 50ms; SDNN = Standard deviation of interval between two normal R-peaks, LF/HF Ratio = Ration of relative power of the low-frequency band (0.04–0.15Hz) to relative power of the high-frequency band (0.15–0.4Hz).
